# Vitamin D deficiency and risk of cardiovascular diseases: a narrative review

**DOI:** 10.1186/s40885-018-0094-4

**Published:** 2018-06-22

**Authors:** Babikir Kheiri, Ahmed Abdalla, Mohammed Osman, Sahar Ahmed, Mustafa Hassan, Ghassan Bachuwa

**Affiliations:** Department of Internal Medicine, Hurley Medical Center/Michigan State University, Two Hurley Plaza, Suite 212, Flint, MI 48503 USA

**Keywords:** Vitamin D, Cholecalciferol, Cardiovascular disease, Hypertension, Blood pressure, Coronary artery disease, Myocardial infarction, Ischemic heart disease, Stroke, Vitamin D supplementation

## Abstract

Vitamin D, a fat-soluble prohormone, has wide-ranging roles in the regulation of many physiological processes through their interactions with the vitamin D receptors (VDR). It plays a major role in bones and calcium metabolism. Vitamin D deficiency is not uncommon and it has been associated with many health-related issues, including skeletal and non-skeletal complications. The association of low vitamin D and cardiovascular diseases and risk factors has been explored in both animal and human studies. However, studies and trials on the effect of vitamin D supplementation on cardiovascular risk factors and hypertension are conflicting with inconsistent results. Therefore, large, well-powered randomized controlled trials are warranted. If successful, supplementation with easy and low-cost vitamin D can impact our health positively. Here, we summarized the evidence for the association of vitamin D, cardiovascular diseases and risk factors, including coronary artery diseases, stroke, and hypertension, and mortality, with special consideration to resistant hypertension.

## Background

Vitamin D is metabolized by hepatic 25-hydroxylase then renal 1a-hydroxylase into its active form, calcitriol, which exerts its function on the vitamin D receptor (VDR) in nearly 30 different tissues [1]. Most of the nutritional requirements of vitamin D are derived from cutaneous solar ultraviolet radiation (80–100%) [2] and to a lesser extent from foods naturally containing or fortified with vitamin D [3]. The best measurement for vitamin D status is its metabolite 25-hydroxyvitamin D (25[OH]D) level [1, 4].

Vitamin D deficiency has been linked to several health outcomes [5], including musculoskeletal (rickets, bone fractures, osteomalacia, osteopenia, osteoporosis and muscle weakness) [3] and non-skeletal complications [6]. Non-skeletal complications include cardiovascular diseases and risk factors [7, 8] such as congestive heart failure [9], impaired systolic and diastolic function [10], myocardial infarction [11], peripheral vascular disease [12], abdominal aortic aneurysm in older men [13], nonvalvular AF [14, 15] and hypertension [16]. In addition, it was also associated with tuberculosis, rheumatoid arthritis, multiple sclerosis, inflammatory bowel diseases, cancers [1], schizophrenia [2], depression, cognitive deficits [17], common obesity [18], non-alcoholic fatty liver disease [19], cystic fibrosis [20], burn injuries [21], type 1 diabetes [4], type 2 diabetes [22, 23], insulin resistance and metabolic syndrome [24–26].

In this narrative review, we aimed to summarize the evidence for the association of vitamin D deficiency with cardiovascular diseases and risk factors, including coronary artery diseases, stroke, and hypertension.

### Epidemiology

Vitamin D deficiency is widespread, the lowest vitamin D levels are commonly found in regions such as the Middle East and South Asia and the main risk factors were attributed to elderly women, higher latitude, winter season, less sunlight exposure, skin pigmentation, dietary intake and low vitamin D fortified foods [27]. It was estimated that the prevalence of vitamin D deficiency is approximately 30–50% of the general population [28]. Furthermore, vitamin D deficiency is still common in sunshine countries [29]. In a large Middle Eastern study of 60,979 patients from 136 countries with yearlong sunlight, 82.5% of studied patients were found to have vitamin D insufficiency [30].

There is an epidemic of vitamin D deficiency worldwide, which represents a major factor of many chronic diseases and has led some authors to suggest annual vitamin D measurement coupled with adequate intake and greater awareness of its consequences [4, 31]. In the United States, there was an increasing prevalence of vitamin D deficiency observed from a sample of 18,158 individuals between 1988 and 1994 compared with a sample of 20,289 individuals between 2000 and 2004 with 5–9 nmol/l decrease in vitamin D levels [32].

Vitamin D levels were found to be lowest in Blacks, followed by Hispanics and Chinese, and adequate in Whites (Multi-Ethnic Study of Atherosclerosis MESA) [33]. Another study done by Yetley in 2008 demonstrated that non-Hispanic blacks and Mexican Americans tend to have lower levels of vitamin D in comparison with non-Hispanic whites [34]. He also found vitamin D to be significantly lower among obese and non-college educated individuals, as well as those with poor health statuses, hypertension, low high-density lipoprotein levels and low milk consumption. Furthermore, the level of vitamin D deficiency was found to be alarmingly lower in winter and spring in a study done in British adults [35].

### Vitamin D and cardiovascular diseases

Vitamin D deficiency has been linked to several cardiovascular risk factors [36, 37]. Through increased renin and angiotensin II synthesis, vitamin D deficiency can increase the production of reactive oxygen species and G protein RhoA, resulting in inhibition of the pathways necessary for intracellular glucose transporter and thus the development of insulin resistance and metabolic syndrome [25]. In addition, direct effects of vitamin D upon smooth muscle calcification and proliferation could contribute to their effects on cardiovascular health [38]. In the Inter99 study of 6784 individuals, high vitamin D level was associated with a favorable lipid profile and lower incidence of metabolic syndrome [39].

Furthermore, in an analysis of NHANES III 1988–1994, low vitamin D was associated with cardiovascular disease (CVD) [7] and select CVD risk factors, including diabetes mellitus (DM), obesity, and hypertriglyceridemia [24]. In a prospective nested case-control study between 1993 and 1999 of 18,225 US men (Health Professionals Follow-Up Study), low vitamin D was associated with a higher risk of myocardial infarction in comparison with sufficient 25(OH)D after multivariate adjustment [11]. Kim and colleagues have found a high prevalence of hypovitaminosis D in individuals with cardiovascular diseases, namely coronary heart disease and heart failure, after controlling for age, race and gender, using data from NHANES 2001–2004 [8].

Additional prospective study of the Integrated Intermountain Healthcare system database of 41,504 patients has shown an association between vitamin D deficiency and an increase in the prevalence of DM, HTN, hyperlipidemia, and peripheral vascular disease (PVD) (*P* < 0.0001) as well as with incident death, heart failure, coronary artery disease/myocardial infarction, stroke and their composite [40]. Also, low serum 25(OH)D was identified as casually associated with increased risk for CVD on the basis of Hill’s criteria for causality in a biological system [41]. In a meta-analysis of 19 prospective studies in 65,994 participants, Wang et al. have demonstrated a linear and inverse association between circulating vitamin D level and risk of cardiovascular diseases [42].

### Vitamin D deficiency and coronary artery disease

The association of vitamin D deficiency with coronary artery diseases (CADs) have been investigated in many studies [43–45]. In 1978, a Danish study found that low vitamin D levels were significantly associated with angina and myocardial infarction [46]. In a multicenter US cohort study evaluating patients admitted with acute coronary syndrome (ACS), about 95% of patients were found to have low vitamin D levels [47]. In a study conducted by Dziedzie et al., low vitamin D levels were observed in patients with myocardial infarction history [48]. In a case-control study (*n* = 240), Roy et al. reported that severe vitamin D deficiency was associated with increased risk of acute myocardial infarction after adjusting for risk factors [49]. Similar findings were reported from Health Professionals Follow-up Study which included 18,225 participants. In this study, at 10-year follow-up, participants with normal vitamin D level had about half the risk of myocardial infarction [11]. In a large prospective study (*n* = 10,170), low vitamin D levels were found to be associated with increased risk of ischemic heart disease, myocardial infarction, and early death during 9 years of follow-up [50]. Additionally, in a meta-analysis of 18 studies, low vitamin D levels were found to have an increased risk of ischemic heart disease and early death [50].

### Vitamin D and hypertension

Hypertension is the most common presentation to primary care providers [51] and represents a major chronic health disease in developed countries [52]. The prevalence of hypertension in adults is approximately 29% [53] with an estimated 1.6 billion cases of hypertension expected in 2025 [54].

### Pathophysiology

It is hypothesized that vitamin D deficiency increases blood pressure through the renin-angiotensin system. Earlier animal studies demonstrated that vitamin D receptor-null (VDR-null) mice have a several-fold increase in renin expression and plasma angiotensin II production, which leads to hypertension, cardiac hypertrophy and increased water intake. In addition, renin suppression was observed in wild-type mice after 1,25(OH)2D3 injection. Therefore, 1,25(OH)2D3 was considered a novel negative endocrine regulator of the renin-angiotensin axis [55]. A later study showed profound heart hypertrophy in vitamin D receptor knockout (VDR-KO) mice, which suggested direct blunting of cardiomyocyte hypertrophy by calcitriol [56]. Through a central antioxidative mechanism, 1,25(OH)2D3 has normalized overactivation of the central renin-angiotensin system in 1-alpha-hydroxylase knockout mice [57]. Furthermore, using mouse models, the elimination of VDR in vascular endothelial cells resulted in a reduction of endothelial nitric oxide synthase expression and impaired endothelial relaxation [58].

In 2011, a study conducted by Argacha et al. revealed that vitamin D-deficient male rats have increased systolic blood pressure, superoxide anion production, angiotensin II and atrial natriuretic peptide with observed changes in 51 cardiac gene expressions important in the regulation of oxidative stress and myocardial hypertrophy [59]. Also, another study of vitamin D-deficient mice showed increased systolic blood pressure, diastolic blood pressure, high plasma renin-angiotensin activity and reduced urinary sodium excretion, which was reversed after 6 weeks of a vitamin D-sufficient chow diet [60]. In the same study, vitamin D-deficient mice on a high-fat diet had increased atherosclerosis in their aorta with increased macrophage infiltration, fat deposition, and endoplasmic reticulum stress activation. These results indicate vitamin D deficiency is associated with the development of hypertension and accelerated atherosclerosis [60]. In another study on double-transgenic rats, vitamin D-depleted rats were shown to exacerbate hypertension (HTN) and impact the renin-angiotensin system, which can contribute to end-organ damage [61].

For the first time in humans, a prospective cohort study of 3316 patients (1997–2000) in southwest Ludwigshafen (Ludwigshafen Risk and Cardiovascular Health LURIC Study) showed a steady increase of plasma renin concentration with declining levels of 25(OH)D and 1,25(OH)2D, as well as a similar increase in angiotensin 2 [62]. Another study showed increased renin-angiotensin system activity in obese hypertensive individuals with low 25(OH)D [63]. Furthermore, another study, which included 375 hypertensive and 146 normotensive individuals, showed that genetic variation at the Fok1 polymorphism of the vitamin D receptor gene and 25(OH)D levels were associated with plasma renin activity in hypertension, a finding that supports the vitamin D-VDR complex as a renin regulator in humans [64]. Therefore, vitamin D analogs have been suggested to be used as renin inhibitors similar to ACE inhibitors and ARBs for patients with hyperreninemia, which can benefit patients with metabolic syndrome and/or hypertension [25]. Other mechanisms that can lead to hypertension in vitamin D-deficient patients are arterial stiffness [65, 66], endothelial dysfunction [67], and hyperparathyroidism [68].

### Studies regarding vitamin D and hypertension

There is accumulating evidence for the association between vitamin D and blood pressure. An earlier analysis of NHANES III 1988–1994 of 12,644 participants aged > 20 years showed an inverse association between vitamin D level and blood pressure [69]. Similar results were obtained from analysis of NHANES 2003–2006 of 7228 participants [70], the Insulin Resistance Atherosclerosis Family Study (IReSFS) [71], and the Kaiser Permanente Southern California health plan [72].

Forman and colleagues have also demonstrated an inverse association between vitamin D and risk of incident hypertension from two prospective cohort studies including 613 (followed for 4–8 years) and 38,388 (followed for 16–18 years) men from the Health Professionals’ Follow-Up Study and 1198 (followed for 4–8 years) and 77,531 (followed for 16–18 years) women from the Nurses’ Health Study. Their results, combining men and women with measured 25(OH)D levels, showed a pooled relative risk of 3.18 (95% confidence interval [CI] 1.39–7.29) [73].

Worldwide studies have also demonstrated such an association. In a cross-sectional study of 833 Caucasian males in Uppsala (central Sweden), a threefold higher prevalence of confirmed hypertension was found in participants with 25(OH)D levels < 37.5 nmol/L [74]. Additionally, a cross-sectional analysis of 1460 participants in Shanghai showed high prevalence of vitamin D deficiency (55.8%) in middle-aged and elderly Chinese men [75]. In adolescents (aged 13–15), a study of 1441 Peruvians showed an inverse association between vitamin D deficiency and blood pressure, which may predispose risk of HTN later in adulthood [76].

### Vitamin D and aging related cardiovascular disease and hypertension

Older adults are at increased risk for vitamin D deficiency, largely due to reduced vitamin D intake and decreased cutaneous synthesis [77, 78]. Beyond skeletal health, accumulated evidence has linked vitamin D deficiency to cardiovascular diseases and hypertension in older patients. Advancing age is associated with increased cardiovascular diseases due to vascular endothelial dysfunction as indicated by decreased peripheral arterial endothelium-dependent dilatation [79]. The mechanisms underpinning this association have been attributed mainly to the reductions in nitric oxide synthesis and increases in oxidative stress with aging [79]. Furthermore, advancing age is associated with reduced blood vessels walls compliance and increased incidence of hypertension [80]. Vitamin D deficiency has been found to modulate the vascular endothelial function with aging [79] and, therefore, increase the incidence of hypertension. In a study conducted by Kestenbaum et al., 2312 older participants (≥ 65 years) without cardiovascular disease at baseline were followed for a median period of 14 years [81]. Their results showed that low 25(OH)D was associated with incident cardiovascular disease and mortality. Furthermore, in a cross-sectional study conducted by Dorjgochoo et al., low 25(OH)D levels were associated with hypertension among older adults [75].

### Vitamin D and resistant hypertension

Resistant hypertension is an increasingly common health problem and considered as a strong risk factor cardiovascular disease [82]. It is defined as any blood pressure above the target despite adherence to three antihypertensive agents, including a diuretic, with optimal doses or the use of at least four antihypertensive agents regardless of the blood pressure level [83, 84]. Over the past 2 decades, the prevalence of resistant hypertension has almost doubled from 5.5% in 1988–1994 to 11.8% in 2005–2008 [82]. Many factors have been attributed to resistant hypertension such as obesity and excessive adipose tissue as well as hyperaldosteronism [85]. Low vitamin D was linked to resistant hypertension secondary to increased adiposity and metabolic disturbances, including insulin resistance [86]. Furthermore, vitamin D deficiency was found to be associated with increased aldosterone levels [87].

Several studies have demonstrated the relation between vitamin D and resistant hypertension. In a study of 150 patients, lower vitamin D level was associated with resistant hypertension [88]. Additionally, in a study of patients with resistant hypertension (*N* = 101) who underwent renal sympathetic denervation (RD), low vitamin D was associated with a decreased systolic blood pressure response to RD [89].

### Vitamin D and cerebrovascular accident

Cerebrovascular accident (CVA) is the most devastating neurological conditions which can cause physical impairment and even death. Accumulating evidence suggests that vitamin D deficiency is associated with increased risk of CVA [90]. The underlining mechanisms have been largely attributed to the association of vitamin D with cardiovascular risk factors such as hypertension and DM. In addition, epidemiological studies have suggested that vitamin D deficiency is an independent risk factor for CVA [42]. In a study conducted by Sun et al. (*n* = 464), low vitamin D levels were associated with increased risk of developing CVA in comparison with high levels [91]. In the Reasons for Geographic and Racial Differences in Stroke (REGARDS) study, vitamin D deficiency was found to be a risk factor for incident CVA unrelated to race [92]. Furthermore, vitamin D level was found to be a predictor of both severity at admission and favorable functional outcome in patients with ischemic CVA [90].

### Vitamin D and mortality

In a prospective cohort study of 3258 patients in southwest Germany (Cardiac Center Ludwigshafen) with a median follow-up of 7.7 years showed that low vitamin D level is independently associated with higher all-cause mortality (HR 2.08; 95% CI 1.60–2.70) and cardiovascular mortality (HR 2.22; 95% CI 1.57–3.13) [93]. Additionally, in the Uppsala Longitudinal Study of Adult Men of 1194 elderly men, both low and high serum 25(OH)D levels were associated with increased risk of overall and cancer mortality, however, only low level was associated with cardiovascular mortality [94]. In Finland, a study of 1136 participants from Kuopio Ischaemic Heart Disease Risk Factor (KIHD) Study showed that vitamin D deficiency was associated with a higher risk of death [95].

The rate of all-cause mortality of 13,331 adults > 19 years from the NHANES III Linked Mortality Files (1988–994) was independently higher by 26% for individuals with low vitamin D levels (25[OH]D < 17.8 ng/ml) compared to the highest quartile [96]. Additionally, in a sample of 3408 individuals aged > 64, low baseline 25(OH)D levels were associated with increased all-cause mortality risk after adjusting for demographics, season, and cardiovascular risk factors (hazard ratio 0.95; 95% CI 0.92–0.98, per 10 nmol/L 25[OH]D) [97]. A similar result was obtained from NHANES 2001–2004 analysis with an increase in all-cause and CVD mortality [98, 99]. A large meta-analysis of 8 prospective cohort studies across the US and Europe of 26,018 individuals showed a remarkable consistency of the association between 25(OH)D level and all-cause and cause-specific mortality [100]. Additionally, a meta-analysis found a nonlinear decrease in mortality with increasing 25(OH)D levels in 14 prospective cohort studies (*n* = 62,548) [101].

### Management

#### Vitamin D supplementation

Multiple studies were done to evaluate the effect of Vitamin D supplementation on cardiovascular disease and mortality. In a randomized clinical trial of 5108 community-residents adults aged 50–84 year, monthly high-dose vitamin D supplementation (100,000 IU) did not prevent cardiovascular disease compared with placebo [102]. Furthermore, the EVITA (Effect of Vitamin D on All-cause Mortality in Heart Failure) randomized trial, did not show a reduction of mortality in patients with advanced heart failure received a daily vitamin D of 4000 IU compared with placebo [103]. Also, in a systematic review and meta-analysis of 18 trials and 13 observational studies, there were uncertain associations between vitamin D status and cardiometabolic outcomes [104]. In addition, another meta-analysis by Wang et al. showed a linear inverse association between 25(OH)D and risk of CVD [105]. While Ford et al.’s meta-analysis showed some benefits of vitamin D supplementation on cardiac failure, it did not show benefits on myocardial infarction/stroke [106].

With the proven association between vitamin D and hypertension, several studies were conducted to see whether vitamin D supplementation would help in treating hypertension. However, these studies resulted in different outcomes and recommendations. Some studies have shown some beneficial outcomes with vitamin D supplementation in reducing blood pressure in patients with low baseline vitamin D levels [39, 106–109]. In a study of 112 patients conducted in Denmark, a 20 weeks’ supply of 3000 IU cholecalciferol in winter resulted in a nonsignificant reduction of 3/1 mmHg (P 0.26/0.18), however, significant results were obtained in patients with low baseline 25(OH)D (< 32 ng/ml) of 4/3 mmHg (P 0.05/0.01) [108]. Another study targeted females over 69 years old showed the benefit of 8 weeks’ supplementation of vitamin D3 (800 IU) and calcium (1200 mg) on systolic blood pressure by a 5 mmHg or more decrease in SBP in 60 subjects (81%) (*P* = 0.04) [107]. In a randomized controlled trial of 283 African Americans between 2008 and 2010 showed for each 1 ng/ml increase in 25(OH)D there was a 0.2 mmHg reduction in SBP (*P* = 0.02) after 3 months’ supplementation of (doses 1000, 2000 or 4000 IU) cholecalciferol [106]. On the other hand, some studies did not show any reduction in blood pressure with vitamin D supplementation [110–112]. As we see in a randomized controlled trial of 161 predominately white individuals, large doses of vitamin D3 (200,000 for 2 months then 100,000 monthly) up to 18 months showed no effect on BP [111]. Also, the DAYLIGHT randomized controlled trial showed no benefit of vitamin D supplementation on BP [112]. A similar result was found in Austria in the Styrian Vitamin D Hypertension Trial (2011–2014) of 200 participants after 8 weeks of vitamin D3 2800 IU [110].

Therefore, multiple meta-analyses were conducted to study the benefit of replacing vitamin D in hypertensive patients. But again, these meta-analyses also had mixed results. Beveridge et al. did a meta-analysis of 46 trials and concluded there was no effect of vitamin D supplementation on blood pressure [113]. A meta-analysis done by Wu et al. of 8 randomized controlled trials studying the effect of calcium and vitamin D supplementation on blood pressure showed no meaningful effect on daytime office BP [114]. Furthermore, two systemic reviews and meta-analyses attributed the inconsistency in evidence regarding vitamin D supplementation’s effect on blood pressure to the heterogeneity in study design [115, 116]. However, in a mendelian randomization study, Vimaleswaran et al. have found a genetic evidence that increased vitamin D concentrations are causally associated with reduced blood pressure and the risk of hypertension [117].

With regards to the efficacy of vitamin D supplementation on reducing CVA, available evidence are conflicting [118]. However, in a recent small scale randomized clinical trial, a single dose of 6 lac IU of Cholecalciferol Intramuscular (IM) injection was associated with a significant improvement in the stroke outcome after three months [119]. We hope that the ongoing Vitamin D and Omega-3 Trial (VITAL) would shed some light on the role of vitamin D supplementation in reducing cardiovascular events, including CVA [120, 121].

With the above mentioned evidence, the inconsistencies between those studies could be due to the differences in vitamin D preparations, follow-up length, patient compliance with the medications, differences in study populations’ baseline characteristics, sample size and the metabolic heterogeneity among the included patients [122]. So, there is a worldwide call for a larger randomized control trial [12, 123–136].

## Conclusions

With high prevalence globally, vitamin D deficiency is not uncommon. It is associated with adverse health-related problems. Current evidence suggests a higher risk of cardiovascular diseases and risk factors with lower vitamin D levels. Furthermore, low vitamin D is associated with hypertension and higher cardiovascular and all-cause mortality. The benefit of vitamin D supplementation to ameliorate the major adverse cardiovascular diseases and hypertension are conflicting with many confounding biases. Therefore, larger randomized clinical trials are warranted to explore the benefits of vitamin D supplementation, which would at least reduce the impact of such high health problems.

## References

[1] Zittermann A (2003). Vitamin D in preventive medicine: are we ignoring the evidence?. Br J Nutr.

[2] Kimlin MG (2008). Geographic location and vitamin D synthesis. Mol Aspects Med.

[3] Holick MF (2007). Vitamin D deficiency. N Engl J Med.

[4] Holick MF (2004). Vitamin D: importance in the prevention of cancers, type 1 diabetes, heart disease, and osteoporosis. Am J Clin Nutr.

[5] Basit S (2013). Vitamin D in health and disease: a literature review. Br J Biomed Sci.

[6] Judd SE, Tangpricha V (2010). Vitamin D Deficiency and Risk for cardiovascular disease. Am J Med Sci.

[7] Kendrick J, Targher G, Smits G, Chonchol M (2009). 25-Hydroxyvitamin D deficiency is independently associated with cardiovascular disease in the third National Health and nutrition examination survey. Atherosclerosis.

[8] Kim D, Sabour S, Sagar U, Adams S, Whellan D (2008). Prevalence of hypovitaminosis D in cardiovascular diseases (from the National Health and nutrition examination survey 2001 to 2004). Am J Cardiol.

[9] Zittermann A, Schleithoff SS, Koerfer R (2006). Vitamin D insufficiency in congestive heart failure: why and what to do about it?. Heart Fail Rev.

[10] Pekkanen MP, Ukkola O, Hedberg P, Piira OP, Lepojärvi S, Lumme J, Tulppo M, Huikuri H (2015). Serum 25-hydroxyvitamin D is associated with major cardiovascular risk factors and cardiac structure and function in patients with coronary artery disease. Nutr Metab Cardiovasc Dis.

[11] Giovannucci E, Liu Y, Hollis B, Rimm E (2008). 25-hydroxyvitamin D and risk of myocardial infarction in men: a prospective study. Arch Intern Med.

[12] McGreevy C, Williams D (2011). New insights about vitamin D and cardiovascular disease: a narrative review. Ann Intern Med.

[13] Wong YYE, Flicker L, Yeap BB, McCaul KA, Hankey GJ, Norman PE (2013). Is hypovitaminosis D associated with abdominal aortic aneurysm, and is there a dose-response relationship?. Eur J Vasc Endovasc Surg.

[14] Demir M, Uyan U, Melek M (2014). The effects of vitamin D deficiency on atrial fibrillation. Clin Appl Thromb Hemost.

[15] Ozcan OU, Gurlek A, Gursoy E, Gerede DM, Erol C (2015). Relation of vitamin D deficiency and new-onset atrial fibrillation among hypertensive patients. J Am Soc Hypertens.

[16] Pavlovic D, Josipovic J, Pavlovic N (2011). Vitamin D and hypertension. Period Biol.

[17] Barnard K, Colón-emeric C (2010). Extraskeletal effects of vitamin D in older adults: cardiovascular disease, mortality, mood, and cognition. Am J Geriatr Pharmacother.

[18] Foss YJ (2009). Vitamin D deficiency is the cause of common obesity. Med Hypotheses.

[19] Küçükazman M, Ata N, Dal K, Yeniova A, Kefeli A, Basyigit S, Aktas B, Akin K, Ağladıoğlu K, Üre Ö, Topal F, Nazligül Y, Beyan E, Ertugrul D (2014). The association of vitamin D deficiency with non-alcoholic fatty liver disease. Clin (Sao Paulo).

[20] Donovan DS, Papadopoulos A, Staron RB, Addesso V, Schulman L, Gregor CMC, Cosman F, Lindsay RL, Shane E (1998). Bone mass and vitamin D deficiency in adults with advanced cystic fibrosis lung disease. Am J Respir Crit Care Med.

[21] Klein GL, Chen TC, Holick MF, Langman CB, Price H, Celis MM, Herndon DN (2004). Synthesis of vitamin D in skin after burns. Lancet.

[22] Liu E, Meigs JB, Pittas AG, Economos CD, Mckeown NM, Booth SL, Jacques PF (2010). Predicted 25-hydroxyvitamin D score and incident type 2 diabetes in the Framingham offspring study. Am J Clin Nutr.

[23] Baz-hecht M, Goldfine AB (2010). The impact of vitamin D deficiency on diabetes and cardiovascular risk. Curr Opin Endocrinol Diabetes Obes.

[24] Martini LA, Wood RJ (2006). Vitamin D status and the metabolic syndrome. Nutr Rev.

[25] Rammos G, Tseke P, Ziakka S (2008). Vitamin D, the renin-angiotensin system, and insulin resistance. Int Urol Nephrol.

[26] Chiu KC, Chu A, Go VLW, Saad MF (2004). Hypovitaminosis D is associated with insulin resistance and beta cell dysfunction. Am J Clin Nutr.

[27] Mithal A, Wahl DA, Burckhardt P, Eisman JA, Fuleihan GE, Josse RG, Lips P, Morales-Torres J (2009). Global vitamin D status and determinants of hypovitaminosis D. Osteoporos Int.

[28] Lee JH, Keefe JHO, Bell D, Hensrud DD, Holick MF (2017). Vitamin D deficiency an important, common, and easily treatable cardiovascular risk factor?. J Am Coll Cardiol.

[29] Prentice A (2008). Vitamin D deficiency: a global perspective. Nutr Rev.

[30] Haq A, Svobodová J, Imran S, Stanford C, Razzaque MS (2016). Journal of Steroid Biochemistry & Molecular Biology Vitamin D de fi ciency: A single centre analysis of patients from 136 countries. J Steroid Biochem Mol Biol.

[31] Zhang R, Naughton DP (2010). Vitamin D in health and disease: current perspectives. Nutr J.

[32] Looker AC, Pfeiffer CM, Lacher DA, Schleicher RL, Picciano F, Yetley EA (2008). Serum 25-hydroxyvitamin D status of the US population: 1988–1994 compared with 2000–2004. Am J Clin Nutr.

[33] Robinson-cohen C, Hoofnagle AN, Ix JH, Kestenbaum R, de Boer IH (2013). Racial differences in the association of serum 25-hydroxyvitamin D concentration with coronary heart disease events. JAMA.

[34] Yetley EA (2008). Assessing the vitamin D status of the US population. Am J Clin Nutr.

[35] Hyppönen E, Power C (2007). Hypovitaminosis D in British adults at age 45 y: nationwide cohort study of dietary and lifestyle predictors. Am J Clin Nutr.

[36] Lavie CJ, Lee JH, Milani RV (2011). Vitamin D and Cardiovascular disease: will it live up to its hype?. J Am Coll Cardiol.

[37] Wang L, Song Y, Manson J, Pilz S, März W, Michaëlsson K, Lundqvist A, Jassal S, Barrett-Connor E, Zhang C, Eaton C, May H, Anderson J, Sesso H (2012). Circulating 25-hydroxy-vitamin D and risk of cardiovascular disease: a meta-analysis of prospective studies. Circ Cardiovasc Qual Outcomes.

[38] Heston TF (2010). Hypovitaminosis D in hypertension. South Med J.

[39] Skaaby T, Nystrup L, Pisinger C, Jørgensen T, Thuesen B, Fenger M, Linneberg A (2012). Vitamin D status and changes in cardiovascular risk factors: a prospective study of a general population. Cardiology.

[40] Anderson JL, May HT, Horne BD, Bair TL, Hall NL, Carlquist JF, Lappé DL, Muhlestein JB (2010). Relation of vitamin D deficiency to cardiovascular risk factors, disease status, and incident events in a general healthcare population. Am J Cardiol.

[41] Weyland PG, Grant WB, Howie-Esquivel J (2014). Does sufficient evidence exist to support a causal association between vitamin D status and cardiovascular disease risk? An assessment using Hill’s criteria for causality. Nutrients.

[42] Pilz S, Tomaschitz A, Drechsler C, Zittermann A, Dekker J, März W (2011). Vitamin D supplementation: a promising approach for the prevention and treatment of strokes. Curr Drug Targets.

[43] Milazzo V, De Metrio M, Cosentino N, Marenzi G, Tremoli E (2017). Vitamin D and acute myocardial infarction. World J Cardiol.

[44] Anderson J, May H, Horne B, Bair T, Hall N, Carlquist J, Lappé D, Muhlestein J (2010). Relation of vitamin D deficiency to cardiovascular risk factors, disease status, and incident events in a general healthcare population. Am J Cardiol.

[45] Aleksova A, Belfiore R, Carriere C, Kassem S, La Carrubba S, Barbati G, Sinagra G (2015). Vitamin D deficiency in patients with acute myocardial infarction: an Italian single-center study. Int J Vitam Nutr Res.

[46] Lund B, Badskjaer J, Lund B, Soerensen O (1978). Vitamin D and ischaemic heart disease. Horm Metab Res.

[47] Lee JH, Gadi R, Spertus JA, Tang F, O’Keefe JH (2011). Prevalence of vitamin D deficiency in patients with acute myocardial infarction. Am J Cardiol.

[48] Dziedzic E, Gąsior J, Pawłowski M, Dąbrowski M (2017). Association of Vitamin D Deficiency and Degree of coronary artery disease in cardiac patients with type 2 diabetes. J Diabetes Res.

[49] Roy A, Lakshmy R, Tarik M, Tandon N, Reddy KS, Prabhakaran D (2015). Independent association of severe vitamin D deficiency as a risk of acute myocardial infarction in Indians. Indian Heart J.

[50] Brøndum-Jacobsen P, Benn M, Jensen GB, Nordestgaard BG (2012). 25-Hydroxyvitamin D levels and risk of ischemic heart disease, myocardial infarction, and early death, Arter. Thromb Vasc Biol.

[51] James P, Oparil S, Carter B, Cushman W, Dennison-Himmelfarb C, Handler J, Lackland D, LeFevre M, MacKenzie T, Ogedegbe O, Smith SJ, Svetkey L, Taler S, Townsend R, Wright JJ, Narva A, Ortiz E (2014). 2014 evidence-based guideline for the management of high blood pressure in adults: report from the panel members appointed to the eighth joint National Committee (JNC 8). JAMA.

[52] Wolf-maier K, Cooper RS, Banegas J, Giampaoli S, Hense H, Joffres M, Kastarinen M, Poulter N, Primatesta P, Rodríguez-Artalejo F, Stegmayr B, Thamm M, Tuomilehto J, Vanuzzo D, Vescio F (2003). Hypertension prevalence and blood pressure levels in 6 European countries, Canada, and the United States. JAMA.

[53] Ong KL, Cheung BMY, Man YB, Lau CP, Lam KSL (2007). Prevalence, awareness, treatment, and control of hypertension among United States adults 1999-2004. Hypertension.

[54] Martini LA, Wood RJ (2008). Vitamin D and blood pressure connection: update on epidemiologic, clinical, and mechanistic evidence. Nutr Rev.

[55] Li L, Yin X, Yao C, Zhu X, Wu X (2012). Vitamin D, parathyroid hormone and their associations with hypertension in a Chinese population. PLoS One.

[56] Simpson RU, Hershey SH, Nibbelink KA (2007). Characterization of heart size and blood pressure in the vitamin D receptor knockout mouse. J Steroid Biochem Mol Biol.

[57] Zhang W, Chen L, Zhang L, Xiao M, Ding J, Goltzman D, Miao D (2015). Administration of exogenous 1,25(OH)2D3 normalizes overactivation of the central renin-angiotensin system in 1α(OH)ase knockout mice. Neurosci Lett.

[58] Ni W, Watts SW, Ng M, Chen S, Glenn DJ, Gardner DG (2014). Elimination of vitamin D receptor in vascular endothelial cells alters vascular function. Hypertension.

[59] Argacha J, Egrise D, Pochet S, Fontaine D, Lefort A, Libert F, Goldman S, van de Borne P, Berkenboom G, Moreno-Reyes R (2011). Vitamin D deficiency-induced hypertension is associated with vascular oxidative stress and altered heart gene expression. J Cardiovasc Pharmacol.

[60] Weng S, Sprague JE, Oh J, Riek AE, Chin K, Garcia M, Bernal-Mizrachi C (2013). Vitamin D deficiency induces high blood pressure and accelerates atherosclerosis in mice. PLoS One.

[61] Andersen LB, Przybyl L, Haase N, Qadri F, Sorensen GL, Fruekilde P, Poglitsch M, Gollasch M, Peters J, Muller DN, Christesen HT, Dechend R (2015). Vitamin D depletion aggravates hypertension and target-organ damage. J Am Heart Assoc.

[62] Tomaschitz A, Pilz S, Ritz E, Grammer T, Drechsler C, Boehm BO, März W (2010). Independent association between 1,25-dihydroxyvitamin D, 25-hydroxyvitamin D and the renin-angiotensin system: The Ludwigshafen Risk and Cardiovascular Health (LURIC) study. Clin Chim Acta.

[63] Vaidya A, Forman JP, Williams JS (2011). Vitamin D and the vascular sensitivity to angiotensin II in obese Caucasians with hypertension. J Hum Hypertens.

[64] Vaidya A, Sun B, Forman JP, Hopkins PN, Brown NJ, Kolatkar NS, Williams GH, Williams JS (2011). The Fok1 vitamin D receptor gene polymorphism is associated with plasma renin activity in Caucasians. Clin Endocrinol (Oxf).

[65] Lee J, Oh S, Ha W, Kwon H, Sohn T, Son H, Cha B (2012). Serum 25-hydroxyvitamin D concentration and arterial stiffness among type 2 diabetes. Diabetes Res Clin Pr.

[66] Kuloğlu O, Gür M, Şeker T, Kalkan G, Şahin D, Tanboğa I, Koyunsever N, Harbaloğlu H, Türkoğlu C, Akyol S, Elbasan Z, Acele A, Caylı M (2013). Serum 25-hydroxyvitamin D level is associated with arterial stiffness, left ventricle hypertrophy, and inflammation in newly diagnosed hypertension. J Invest Med.

[67] Al Mheid I, Patel R, Murrow J, Morris A, Rahman A, Fike L, Kavtaradze N, Uphoff I, Hooper C, Tangpricha V, Alexander RW, Brigham K, Quyyumi AA (2011). Vitamin D status is associated with arterial stiffness and vascular dysfunction in healthy humans. J Am Coll Cardiol.

[68] Giovannucci E (2009). Vitamin D and cardiovascular disease. Curr Atheroscler Rep.

[69] Scragg R, Sowers M, Bell C (2007). Serum 25-hydroxyvitamin D, ethnicity, and blood pressure in the third National Health and nutrition examination survey. Am J Hypertens.

[70] Zhao G, Ford ES, Li C, Kris-etherton PM, Etherton TD, Balluz LS (2010). Independent associations of serum concentrations of 25-hydroxyvitamin D and parathyroid hormone with blood pressure among US adults. J Hypertens.

[71] Schmitz KJ, Skinner HG, Bautista LE, Fingerlin TE, Langefeld CD, Hicks PJ, Haffner SM, Bryer-ash M, Wagenknecht LE, Bowden DW, Norris JM, Engelman CD (2009). Association of 25-hydroxyvitamin D with blood pressure in predominantly 25-hydroxyvitamin D deficient Hispanic and African Americans. Am J Hypertens.

[72] Bhandari S, Pashayan S, Liu I, Rasgon S, Kujubu D, Tom T, Sim JJ (2011). 25-hydroxyvitamin D levels and hypertension rates. J Clin Hypertens (Greenwich).

[73] Forman JP, Giovannucci E, Holmes MD, Bischoff-ferrari HA, Tworoger SS, Willett WC, Curhan GC (2007). Plasma 25-hydroxyvitamin D levels and risk of incident hypertension. Hypertension.

[74] Burgaz A, Byberg L, Rautiainen S, Orsini N, Håkansson N, Arnlöv J, Sundström J, Lind L, Melhus H, Michaëlsson K, Wolk A (2011). Confirmed hypertension and plasma 25(OH)D concentrations amongst elderly men. J Intern Med.

[75] Dorjgochoo T, Shu XO, Xiang Y, Yang G, Cai Q, Li H, Ji B, Cai H, Gao Y, Zheng W (2012). Circulating 25-hydroxyvitamin D levels in relation to blood pressure parameters and hypertension in the shanghai Women’s and Men’s health studies. Br J Nutr.

[76] Tomaino K, Romero KM, Robinson CL, Baumann LM, Hansel NN, Pollard SL, Gilman RH, Mougey E, Lima JJ, Checkley W (2015). Association between serum 25-Hydroxy vitamin D levels and blood pressure among adolescents in two resource-limited settings in Peru. Am J Hypertens.

[77] Meehan M, Penckofer S (2014). The role of vitamin D in the aging adult. J Aging Gerontol.

[78] Mosekilde L (2005). Vitamin D and the elderly. Clin Endocrinol (Oxf).

[79] Seals DR, Jablonski KL, Donato AJ (2011). Aging and vascular endothelial function in humans. Clin Sci.

[80] Boucher BJ (2012). The problems of vitamin d insufficiency in older people. Aging Dis.

[81] Kestenbaum B, Katz R, De Boer I, Hoofnagle A, Sarnak MJ, Shlipak MG, Jenny NS, Siscovick DS (2011). Vitamin D, parathyroid hormone, and cardiovascular events among older adults. J Am Coll Cardiol.

[82] Kumar N, Calhoun D, Dudenbostel T (2013). Management of patients with resistant hypertension: current treatment options. Integr Blood Press Control.

[83] Mancia G, Fagard R, Narkiewicz K, Redón J, Zanchetti A, Böhm M, Christiaens T, Cifkova R, De Backer G, Dominiczak A, Galderisi M, Grobbee D, Jaarsma T, Kirchhof P, Kjeldsen S, Laurent S, Manolis A, Nilsson P, Ruilope L, Schmieder R, Sirnes P, Sleight P, Viigimaa M, Waeber B, Zannad F (2013). 2013 ESH/ESC guidelines for the management of arterial hypertension: the task force for the management of arterial hypertension of the European Society of Hypertension (ESH) and of the European Society of Cardiology (ESC). J Hypertens.

[84] Calhoun DA, Jones D, Textor S, Goff DC, Murphy TP, Toto RD, White A, Cushman WC, White W, Sica D, Ferdinand K, Giles TD, Falkner B, Carey RM (2008). Resistant hypertension: diagnosis, evaluation, and treatment: a scientific statement from the American Heart Association professional education Committee of the Council for high blood pressure research. Circulation.

[85] Zoccali C, Mallamaci F (2014). Does a vitamin D boost help in resistant hypertension control?. Hypertension.

[86] Beydoun MA, Boueiz A, Shroff MR, Beydoun HA, Wang Y, Zonderman AB (2010). Associations among 25-hydroxyvitamin D, diet quality, and metabolic disturbance differ by adiposity in adults in the United States. J Clin Endocrinol Metab.

[87] Rossi GP, Ragazzo F, Seccia TM, Maniero C, Barisa M, Calò LA, Frigo AC, Fassina A, Pessina AC (2012). Hyperparathyroidism can be useful in the identification of primary aldosteronism due to aldosterone-producing adenoma. Hypertension.

[88] Belen E, Şahin İ, Güngör B, Ayça B, Avcı İ, Avşar M, Yıldız S, Akın F, Bozbeyoglu E, Okuyan E (2016). Assessment of 25-Hydroxyvitamin D levels in patients with resistant hypertension. Med Princ Pr.

[89] Pöss J, Mahfoud F, Ukena C, Esler M, Schlaich M, Hering D, Cremers B, Laufs U, Böhm M (2014). Association of vitamin D status and blood pressure response after renal denervation. Clin Res Cardiol.

[90] Wang Y, Ji H, Tong Y, Zhang Z (2014). Prognostic value of serum 25-hydroxyvitamin D in patients with stroke. Neurochem Res.

[91] Sun Q, Pan A, Hu F, Manson J, Rexrode KM (2012). 25-Hydroxyvitamin D levels and the risk of stroke: a prospective study and meta-analysis. Stroke.

[92] Judd S, Morgan C, Panwar B, Howard V, Wadley V, Jenny N, Kissela B, Gutiérrez O (2016). Vitamin D deficiency and incident stroke risk in community-living black and white adults. Int J Stroke.

[93] Dobnig H, Pilz S, Scharnagl H, Renner W, Seelhorst U, Wellnitz B, Kinkeldei J, Boehm B, Weihrauch G, Maerz W (2008). Independent association of low serum 25-hydroxyvitamin d and 1,25-dihydroxyvitamin d levels with all-cause and cardiovascular mortality. Arch Intern Med.

[94] Michaëlsson K, Baron JA, Snellman G, Gedeborg R, Byberg L, Sundstro J, Berglund L, Arnlöv J, Hellman P, Blomhoff R, Wolk A, Garmo H, Holmberg L, Melhus H (2010). Plasma vitamin D and mortality in older men: a community-based prospective cohort study. Am J Clin Nutr.

[95] Virtanen JK, Nurmi T, Voutilainen S, Mursu J, Tuomainen T-P (2011). Association of serum 25-hydroxyvitamin D with the risk of death in a general older population in Finland. Eur J Nutr.

[96] Melamed ML, Michos ED, Post W, Astor B (2008). 25-hydroxyvitamin D levels and the risk of mortality in the general population. Arch Intern Med.

[97] Ginde AA, Scragg ÃR, Schwartz RS, Camargo CA (2009). Prospective study of serum 25-hydroxyvitamin D level, cardiovascular disease mortality, and all-cause mortality in older U.S. adults. J Am Geriatr Soc.

[98] Zhaoa G, Forda ES, Lib C, Croft JB (2012). Serum 25-hydroxyvitamin D levels and all-cause and cardiovascular disease mortality among US adults with hypertension: the NHANES linked mortality study. J Hypertens.

[99] Amer M, Qayyum R (2013). Relationship between 25-hydroxyvitamin D and all-cause and cardiovascular disease mortality. Am J Med.

[100] Schöttker B, Jorde R, Peasey A, Thorand B, Jansen E, Groot L, Streppel M, Gardiner J, Ordóñez-Mena J, Perna L, Wilsgaard T, Rathmann W, Feskens E, Kampman E, Siganos G, Njølstad I, Mathiesen E, Kubínová R, Pająk A, Topor-Madry R, Tamosiunas A, Hughes M, Kee F, Bobak M, Trichopoulou A, Boffetta P, Brenner H (2014). Vitamin D and mortality: meta-analysis of individual participant data from a large consortium of cohort studies from Europe and the United States. BMJ.

[101] Zittermann A, Iodice S, Pilz S, Grant WB, Bagnardi V, Gandini S (2012). Vitamin D deficiency and mortality risk in the general population: a meta-analysis of prospective cohort studies. Am J Clin Nutr.

[102] Scragg R, Stewart AW, Waayer D, Lawes CMM, Toop L, Sluyter J, Murphy J, Khaw K-T, Camargo CA (2017). Effect of monthly high-dose vitamin D supplementation on cardiovascular disease in the vitamin D assessment study: a randomized clinical trial. JAMA Cardiol.

[103] Zittermann A, Ernst JB, Prokop S, Fuchs U, Dreier J, Kuhn J, Knabbe C, Birschmann I, Schulz U, Berthold HK, Pilz S, Gouni-Berthold I, Gummert JF, Dittrich M, Börgermann J (2017). Effect of vitamin D on all-cause mortality in heart failure (EVITA): a 3-year randomized clinical trial with 4000 IU vitamin D daily. Eur Heart J.

[104] Pittas AG, Chung M, Trikalinos T, Mitri J, Brendel M, Patel K, Lichtenstein AH (2010). Systematic review: vitamin D and cardiometabolic outcomes. Ann Intern Med.

[105] Wang L, Song Y, Manson J, Pilz S, März W, Michaëlsson K, Lundqvist A, Jassal S, Barrett-Connor E, Zhang C, Eaton C, May H, Anderson J, Sesso H (2012). Circulating levels of 25Hydroxy-vitamin D and risk of cardiovascular disease: a meta-analysis of prospective studies. Circ Cardiovasc Qual Outcomes.

[106] Forman JP, Scott JB, Ng K, Drake BF, Suarez EG, Hayden DL, Bennett GG, Chandler PD, Hollis BW, Emmons KM, Giovannucci EL, Fuchs CS, Chan AT (2013). Effect of vitamin D supplementation on blood pressure in African-Americans. Hypertension.

[107] Pfeifer M, Begerow B, Minne HW, Nachtigall D, Hansen C (2001). Effects of a short-term vitamin D(3) and calcium supplementation on blood pressure and parathyroid hormone levels in elderly women. J Clin Endocrinol Metab.

[108] Larsen T, Mose FH, Bech JN, Hansen AB, Pedersen EB (2012). Effect of cholecalciferol supplementation during winter months in patients with hypertension: a randomized, placebo-controlled trial. Am J Hypertens.

[109] Cozzolino M, Stucchi A, Rizzo MA, Soldati L, Cusi D, Ciceri P, Brenna I, Elli F, Gallieni M (2012). Vitamin D receptor activation and prevention of arterial ageing. Nutr Metab Cardiovasc Dis.

[110] Pilz S, Gaksch M, Kienreich K, Grübler M, Verheyen N, Fahrleitner-pammer A, Treiber G, Drechsler C, Hartaigh B, Obermayer-pietsch B, Schwetz V, Aberer F, Mader J, Scharnagl H, Meinitzer A, Lerchbaum E, Dekker JM, Zittermann A, März W, Tomaschitz A (2015). Effects of vitamin D on blood pressure and cardiovascular risk factors: a randomized controlled trial. Hypertension.

[111] Scragg R, Slow S, Stewart AW, Jennings LC, Chambers ST, Priest PC, Florkowski CM, Jr CAC, Murdoch DR (2014). Long-term high-dose vitamin D3 supplementation and blood pressure in healthy adults: a randomized controlled trial. Hypertension.

[112] Arora P, Song Y, Dusek J, Plotnikoff G, Sabatine MS, Cheng S, Valcour A, Swales H, Taylor B, Carney E, Guanaga D, Young JR, Karol C, Torre M, Azzahir A, Strachan SM, Neill DCO, Wolf M, Harrell F, Newton-cheh C, Wang TJ (2015). Vitamin D therapy in individuals with prehypertension or hypertension: the DAYLIGHT trial. Circulation.

[113] Beveridge LA, Struthers AD, Khan F, Jorde R, Scragg R, Macdonald HM, Alvarez JA, Boxer RS, Dalbeni A, Gepner AD, Isbel NM, Larsen T, Nagpal J, Petchey WG, Stricker H, Strobel F, Tangpricha V, Toxqui L, Vaquero M, Wamberg L, Zittermann A, Witham M (2015). Effect of vitamin D supplementation on blood pressure: a systematic review and meta-analysis incorporating individual patient data. JAMA Intern Med.

[114] Wu L, Sun D (2017). Effects of calcium plus vitamin D supplementation on blood pressure: a systematic review and meta-analysis of randomized controlled trials. J Hum Hypertens.

[115] Upala S, Sanguankeo A, Congrete S (2016). Effect of cholecalciferol supplementation on arterial stiffness: a systematic review and meta-analysis. Scand Cardiovasc J.

[116] Rodrıguez AJ, Scott D, Srikanth V, Ebeling P (2016). Effect of vitamin D supplementation on measures of arterial stiffness: a systematic review and meta-analysis of randomized controlled trials. Clin Endocrinol (Oxf).

[117] Vimaleswaran KS, Cavadino A, Berry DJ, Power C, Hyppönen E, Jorde R, Grimnes G, Dieff enbach AK, Schöttker B, Saum KU, Brenner H, Lu C, Järvelin MR, Tzoulaki I, Heerspink HJL, Nolte IM, Snieder H, van der Most PJ, Stolk RP, Hartman CA, de Boer RA, van der Harst P, Navis G, de Borst MH, Tikkanen E, Eriksson J, Lorentzon M, Mellström D, Ohlsson C, Wong A, Hardy R, Kuh D, Cooper JA, Acharya J, Humphries SE, Hingorani AD, Kumari M, Kivimaki M, Mangino M, Spector TD, Jablonski KA, Houston DK, Kritchevsky SB, Lohman KK, Ahluwalia TS, Sørensen TIA, Pasko D, Frayling TM, Zgaga L, Campbell H, Theodoratou E, Fraser RM, Wilson JF, Rudan I, Price JF, McLachlan S, Vitart V, Navarro P, Huffman JE, Hayward C, Wright AF, Thiering E, Tiesler CMT, Heinrich J, McCarthy MI, Ingelsson E, Cooper C, Arden N, Dupuis J, Herzig KH, Sebert S, Alves AC, Pouta A, Laitinen J, Kleber ME, März W, Jameson K, Osmond C, Raitakari O, Ripatti S, Lahti J, Eriksson JG, Penninx BW, Billings LK, Florez JC, Rejnmark L, Langdahl BL, Paternoster L, Hernandez DG, Byberg L, Michaëlsson K, Hagström E, Melhus H, Ljunggren O, Lind L, Jula A, Polasek O, Salomaa V, Karlsson M, Bandinelli S, Lehtimäki T, Wang TJ, Pilz S, Whittaker JC, Hyppönen E (2014). Association of vitamin D status with arterial blood pressure and hypertension risk: a mendelian randomisation study. Lancet Diabetes Endocrinol.

[118] Mao P, Zhang C, Tang L, Xian Y, Li Y, Wang W, Zhu X, Qiu H, He J, Zhou Y (2013). Effect of calcium or vitamin D supplementation on vascular outcomes: a meta-analysis of randomized controlled trials. Int J Cardiol.

[119] Shuba N, Prakash B (2017). Role of vitamin D in the outcome of ischemic stroke- a randomized controlled trial. J Clin Diagn Res.

[120] Manson J, Bassuk S, Lee I, Cook N, Albert M, Gordon D, Zaharris E, Macfadyen J, Danielson E, Lin J, Zhang S, Buring J (2012). The VITamin D and OmegA-3 TriaL (VITAL): rationale and design of a large randomized controlled trial of vitamin D and marine omega-3 fatty acid supplements for the primary prevention of cancer and cardiovascular disease. Contemp Clin Trials.

[121] Aruna DP, JoAnn EM (2016). Update on the vitamin D and omega-3 trial (VITAL). J Steroid Biochem Mol Biol.

[122] Abbasi F, Feldman D, Caulfield MP, Hantash FM, Reaven GM (2015). Relationship among 25-hydroxyvitamin D concentrations, insulin action, and cardiovascular disease risk in patients with essential hypertension. Am J Hypertens.

[123] Geleijnse JM (2011). Vitamin D and the prevention of hypertension and cardiovascular diseases: a review of the current evidence. Am J Hypertens.

[124] Pilz S, Tomaschitz A, März W, Drechsler C, Ritz E, Zittermann A, Cavalier E, Pieber T, Lappe J, Grant W, Holick M, Dekker J (2011). Vitamin D, cardiovascular disease and mortality. Clin Endocrinol (Oxf).

[125] Lavie CJ, Lee JH, Milani RV (2011). Vitamin D and cardiovascular disease will it live up to its hype?. J Am Coll Cardiol.

[126] Wuerzner G, Burnier M, Waeber B (2012). Should hypertensive patients take vitamin D?. Curr Hypertens Rep.

[127] Brandenburg VM, Vervloet MG, Marx N (2012). The role of vitamin D in cardiovascular disease: from present evidence to future perspectives. Atherosclerosis.

[128] Muscogiuri G, Sorice GP, Ajjan R, Mezza T, Pilz S, Prioletta A, Scragg R, Volpe SL, Witham MD, Giaccari A (2012). Can vitamin D deficiency cause diabetes and cardiovascular diseases? Present evidence and future perspectives. Nutr Metab Cardiovasc Dis.

[129] Rosen CJ, Adams JS, Bikle DD, Black DM, Demay MB, Manson JE, Murad MH, Kovacs CS (2012). The nonskeletal effects of vitamin D: an Endocrine Society scientific statement. Endocr Rev.

[130] Tamez H, Thadhani R (2012). Vitamin D and hypertension: an update and review. Curr Opin Nephrol Hypertens.

[131] Kienreich K, Grübler M, Tomaschitz A, Schmid J, Verheyen N, Rutters F, Dekker JM, Pilz S (2013). Vitamin D, arterial hypertension & cerebrovascular disease. Indian J Med Res.

[132] Liu Z, Woo J, Wu S, Ho SC (2013). The role of vitamin D in blood pressure, endothelial and renal function in postmenopausal women. Nutrients.

[133] Min B (2013). Effects of vitamin d on blood pressure and endothelial function. Korean J Physiol Pharmacol.

[134] Rostand SG (2014). Vitamin D deficiency in the pathogenesis of hypertension: still an unsettled question. Curr Hypertens Rep.

[135] Muscogiuri G, Annweiler C, Duval G, Karras S, Tirabassi G, Salvio G, Balercia G, Kimball S, Kotsa K, Mascitelli L, Pal H, Colao A (2017). Vitamin D and cardiovascular disease: from atherosclerosis to myocardial infarction and stroke. Int J Cardiol.

[136] Wang TJ (2016). Vitamin D and cardiovascular disease. Annu Rev Med.

