# Correction to: Vitamin D deficiency and risk of cardiovascular diseases: a narrative review

**DOI:** 10.1186/s40885-018-0105-5

**Published:** 2018-12-24

**Authors:** Babikir Kheiri, Ahmed Abdalla, Mohammed Osman, Sahar Ahmed, Mustafa Hassan, Ghassan Bachuwa

**Affiliations:** Department of Internal Medicine, Hurley Medical Center/Michigan State University, Two Hurley Plaza, Suite 212, Flint, MI 48503 USA

**Correction to: Clin Hypertens**

**https://doi.org/10.1186/s40885-018-0094-4**

In the original publication of this article [1], the reference number [42] under the section ‘Vitamin D and cardiovascular diseases’ (page No.2) should be [37].
